# Disability and migrants: A double educational challenge for an inclusive and plural school

**DOI:** 10.3389/fpsyg.2022.1017841

**Published:** 2022-11-23

**Authors:** Anna Tataranni

**Affiliations:** Ionian Department of Mediterranean Legal and Economic Systems: Society, Environment, Cultures, Rights, Economies, and Cultures of the Mediterranean, Scientific Disciplinary Field of General and Social Pedagogy (MPED-01), University of Bari “A. Moro”, Bari, Italy

**Keywords:** disability, migrants, inclusion, intercultural and special pedagogy, complex approaches

## Introduction

Migration is a complex phenomenon that affects both the migrant and the country the migrant immigrates to. A migrant lives in a state of precariousness, suspended between the culture of origin and the new country in which they actualize their potential.

When disability concerns are factored in, the issues become even more complicated. This complex relationship requires different levels of reflection where education becomes the promoter of the “double” task inherent in the school and social inclusion of the student with disabilities of immigrant origins. This educational challenge is configured in strongly systemic (Bertallanfy, 1968), ecological (Bronfenbrenner and Morris, 2006) terms and responds to the need to promote the wellbeing of the disabled person in the plurality of their life contexts (school, family, social, etc.), where they require acceptance, respect, and enhancement of their resources and different abilities.

## Dimensions of dual vulnerability and cultural representations

Considering the literature and statistical plans referring to the dual dimensions inherent in disability and migration, it is obvious how risky it is to understand them in their possible intertwining (Leman, 1991; Manço, 2001; Lindsay et al., 2006; Werning et al., 2008).

According to Bochicchio (2018), being a disabled and migrant person involves living in a condition of “double disadvantage with respect to a double plane” of vulnerability and fragility represented by being a migrant and disabled. Martinazzoli (2012) states that double diversity results in double integration fatigue, linked to disability and cultural belonging, conditions that, when added together, can increase discrimination and social exclusion for children and their families (Canevaro et al., 2011; Traina and Caldin, 2014; Friso and Pileri, 2019). Caldin and Dainese (2011), in his studies, highlights how the inclusion of a child with a disability or a child of migrants requires, as a prerequisite, new organizational systems and educational, social, cultural, and political paradigms. He also believes that the situation becomes even more relevant when the condition of disability is combined with that of the migrant since the mournful experience of the parents is determined by the “communication/confirmation of having a disabled child [...] is added that of migration (Caldin, 2012). Pourtois and Desmet (2015) considers families' participation essential to children's learning and inclusion processes. Moro (2001) highlights the importance of knowing the families' life stories, their relationship with their culture of origin, migration path, experience, and the representation of the child's disability.

Today, despite the growth of some dominant knowledge (Western conceptions of law and human rights), there remain great cultural diversities that highlight how the concept of disability varies greatly from one society to another (Scherper-Hughes, 1992; Lepore, 2011a) and from one era to another (Goussot, 2009).

For example, in China and Latin America, disability is regarded as an alteration of energy balance. Gypsy people believe it can be traced to the loss of the soul due to the intrusion of a malignant entity into the healthy body (Picozzi et al., 2005). In Africa, people with albinism are marginalized as they are considered evil and bearers of evil powers (Goussot, 2011). Disability is seen as a punishment, shame, or a form of dishonor: it may cause parents not to embark on any rehabilitation path, and there have been reports of disabled girls being kept in isolation because they are deemed incapable of working and procreating in some parts of Morocco. In the Sérère culture in Senegal, an intellectually disabled person is considered very close to the ancestral world. Moreover, in New Caledonia, the Kanak aborigines consider autistic children an essential social role in the group as they represent a bridge between the living and the dead (Goussot, 2011); the child's particular behavior is seen as a sign of closeness to the deity. In Asian homes, where parents see themselves as villains if their children are not fully dependent on them, and where keeping eye contact is seen as unintelligent, autonomy and maintaining eye contact are seen as inadequate aims.

The all-encompassing nature of culture is demonstrated by the phrase that “each human is shaped and “de-formed” by the skills and abilities they acquire in their social lives as a member of a group” by the observation that the construct of disability, not unlike that of inclusion, is defined by reference to the context and time in which it is realized (Muscarà, 2018).

In Italy, the report “Migrants with Disabilities: Invisible discrimination” by the IDOS Study and Research Center^1^ at the end of 2020 indicates an incidence of about 4% of pupils with disabilities (speech disorders, such as stuttering, reading and writing disorders, hearing impairments, and cognitive delays) (Pini, 2018) of the total number of foreign pupils attending Italian schools.

Because of such varied representations of disability, schools must build neutral meeting spaces free from prejudice and stereotypes to foster cross-cultural encounters and appreciation of differences, listening, dialogue, and reciprocity; it is a matter of fostering dialogue between parents and educators and between them and other figures working in the field (social workers, rehabilitation, welfare, social animators, mediators, etc.) in favor of the child and his family.

To understand the needs of families and especially children with disabilities who are children of migrants, it is necessary to consider what Vygotskij (1986) called the cultural-historical dimension of their development (language, ways of perceiving and thinking, symbolic codes, representation of self and others, education, value system, and deficit representation) (Goussot, 2010) gand to adopt a transcultural perspective and an attitude of cultural decentralization to co-construct bridges between people and cultures, and to create the connections necessary to implement the planning (Martinazzoli, 2012).

## Educational design and methodological approaches for inclusive teaching

When dealing with the problem of immigrant pupils with disabilities, the Italian school is called upon to mobilize resources and skills because school inclusion has been the subject of scientific and educational attention since 1992^2^.

However, the rules (laws, ministerial circulars and guidelines) that accompany inclusion in the perspective of disability^3^ and the reception of migrants^4^ are fragmented: they are not harmonised and integrated with each other. Are not currently present, in the documents that the legislation indicates for pupils with disabilities, today strongly permeated by the ICF approach, (PEI [Individualized Educational Plans] and [PF] Profile of Operation)^5^, which replaces, incorporating them, functional diagnosis and dynamic functional profile), information on the pupil's biography, cultural dimension, language skills, representations of disability by parents/culture of origin, if any, offering an unbalanced view of disability in relation to cultural^6^.

A cross-cultural history, a structured interview with parents that helps them piece together the child's history from the time of the disability diagnosis to the present, as well as their experience with health services and what has been done in the school in the country of origin (Martinazzoli, 2014), and the monograph, a medical-pedagogical dossier that uses the observations of doctors, teachers, and parents to piece together the child's development and schooling (Goussot, 2011).

Co-design is essential because it welcomes, even in progress, information and suggestions in an ecosystemic representation resulting from a bottom-up process that induces the following:

➢ serendipity;
➢ the important sense of not isolating one function from another;
➢ the dysfunctional vs. the isolation of one function; and
➢ the interweaving of functions that are entirely dissimilar to each other.

With co-design, in which cultural mediators, family, school, and health workers are in the field, mutual adaptation develops in a co-evolutionary dynamic for the construction of the common good, where the objectives provide quality, reformulates, support actions, provide new proposal actions and verification, and “contaminates” documentation.

It is also essential to reflect on the design methodologies adopted with regard to educational and didactic interventions, organization, and creating an environment that should respond to the logic of the index for inclusion (Booth and Ainscow, 2002) and universal design for learning (Rose et al., 2005) (UDL); it is also necessary to reflect on the adaptations envisaged to make some intercultural practices accessible (Bolognesi, 2010; Assenza, 2017; Giampaolo and Melacarne, 2017) to all, thus responding to the typical needs of the condition of disability.

To “free” themselves from preconceptions and to bring to life new dimensions of identity, it turns out that training teachers and, by extension, students suitable to the knowledge of other cultures are crucial. Dialogue with the family, a shared worldview, and a significant thread to the child's past and its significance in the present are all crucial components of a well-rounded educational experience. This dialogue should be supported by the language mediator, not just a translator, but a builder of bridges of meaning between different cultural universes.

## Cultural belonging and social context

From the social point of view, it is necessary to examine how disabilities can relate to the notion of humanity and personality defined by local cultures (Ingstad and Whyte, 1995), what the local languages about diversity are, and how people from a certain place behave when faced with people we define as “disabled,” what the migrant family expects, what place disabled people occupy in the society they belong to, who cares for them, what social, relational, and affective opportunities are offered to them, and how the reasons for diversity are interpreted (Lepore, 2011b). What is certain is that families often feel isolated and stigmatized; they are often uninformed about the rights of the disabled and tend not to turn to voluntary associations, a dynamic that deprives them of building supportive relationships.

Pavesi (2017), in this regard, considers it necessary to activate a model of “responsible welfare” that goes beyond the idea of “welfare assistance” to place the person with their needs, abilities, and support networks at the center of intervention. This model is based on making the resources accessible for people, groups, and organizations; it is based on the shared integration of knowledge, professionalism, and resources; it stimulates proximal care of needs by relying on three pillars:

➢ capacitating activation: enhances the resources of people, their networks, and the social context; aims to make them active protagonists of welfare policies and the social project in which they are placed, promoting individual responsibility and self-realization;
➢ shared integration: networking is based on the synergy between public, private, and voluntary associations;
➢ social space of proximity: a physical and symbolic place where the actors involved create a network that can be activated to respond to the needs of the most vulnerable.

Desirable, according to Folgheraiter (2003), is also the creation of a network that integrates the informal dimension (relatives, friends, and neighbors) with the formal dimension (public services and private social services) to enhance both the professional knowledge of health, social, and educational services, as well as the experiential knowledge and relational “closeness” of people close to the migrant family.

In addition, it will be essential to establish connections with other families experiencing similar challenges to create networks of support and mutual aid, as well as to encourage cross-pollination with networks outside the narrow links inherent to disability and cultural affiliation to build a large, efficient network comprised of bridges and mediators.

## Conclusion

To navigate the complexities of the existential dimension and define appropriate interventions to support the development of children with disabilities from the perspective of the life project, it is helpful to take a multidimensional reading of dual diversity that pays attention to multiple domains and elements that are intrinsically connected, such as cultural belonging, disability, educational, family, and social context.

In this context, special pedagogy and intercultural pedagogy should open up to dialogue with other disciplines for a multidimensional reading aimed at implementing interventions that grasp the uniqueness of the person where “I Care” (Don Milani) can guide the different spheres (educational, rehabilitative, social).

There are still many things to accomplish. Although processes of reflection and awareness of the need and urgency to consider the dual condition of diversity have been triggered, there is still a need to explicitly and specifically outline new “guidelines” and new “paradigms” about disability and immigration. It is a matter of effectively orienting all actors in a delicate and complex path of inclusion and integration to inhibit the risk of lack of recognition and fewer social opportunities (Catarci and Fiorucci, 2014).

## Author contributions

The author confirms being the sole contributor of this work and has approved it for publication.

## Conflict of interest

The author declares that the research was conducted in the absence of any commercial or financial relationships that could be construed as a potential conflict of interest.

## Publisher's note

All claims expressed in this article are solely those of the authors and do not necessarily represent those of their affiliated organizations, or those of the publisher, the editors and the reviewers. Any product that may be evaluated in this article, or claim that may be made by its manufacturer, is not guaranteed or endorsed by the publisher.

## Footnotes

^1^ https://www.aziendaisola.it/public/Dossier-Statistico-Immigrazione-2021.pdf

^2^ https://www.gazzettaufficiale.it/eli/id/1992/02/17/092G0108/sg

^3^Legge n. 118 del 30 marzo 1971. Conversione in legge del D.L. 30 gennaio 1971, n. 5 e nuove norme in favore dei mutilati ed invalidi civili. Legge n. 517 del 4 agosto 1977. Norme sulla valutazione degli alunni e sull'abolizione degli esami di riparazione nonché altre norme di modifica dell'ordinamento scolastico. Legge n. 104 del 5 febbraio 1992. Legge-quadro per l'assistenza, l'integrazione sociale e i diritti delle persone handicappate. DPR n. 24 febbraio 1994. Atto di indirizzo e coordinamento relativo ai compiti delle unità sanitarie locali in materia di alunni portatori di handicap. DPR n. 323 del 23 luglio 1998. Regolamento recante Disciplina degli esami di stato conclusivi dei corsi di studio di istruzione secondaria superiore a norma dell'art. 1 della legge 10 dicembre 1997 n. 425. Nota MIUR n. 4798 del 27 luglio 2005. Attività di programmazione dell'integrazione scolastica degli alunni disabili da parte delle Istituzioni scolastiche - Anno scolastico 2005-2006. OM n. 90 del 21 maggio 2001. Norme per lo svolgimento degli scrutini e degli esami nelle scuole statali e non statali di istruzione elementare, media e secondaria superiore - Anno scolastico 2000-2001, in part. l'art. 15. CM n. 125 del 20 luglio 2001. Certificazione per gli alunni in situazione di handicap. DPCM n. 185 del 23 febbraio 2006. Regolamento recante modalità e criteri per l'individuazione dell'alunno come soggetto in situazione di handicap, ai sensi dell'articolo 35, comma 7, della legge 27 dicembre 2002, n. 289. Nota MIUR n. 4274 del 4 agosto 2009. Trasmissione delle “Linee guida per l'integrazione scolastica degli alunni con la disabilità”. D.Lvo n. 66 del 13 aprile 2017. Norme per la promozione dell'inclusione scolastica degli studenti con disabilità, a norma dell'articolo 1, cc. 180 e 181, lettera (c), della legge 13 luglio 2015, n. 107. Nota MIUR n. 1153 del 4 agosto 2017. Chiarimenti in merito alla decorrenza dei termini di applicazione delle indicazioni del D.Lvo n. 66/2017. Nota MIUR n. 15578 del 8 agosto 2017. Chiarimenti in merito alla decorrenza dei termini di applicazione delle indicazioni del D.L. vo n.66/2017. Precisazione. D.Lvo n. 96 del 7 agosto 2019. Disposizioni integrative e correttive al D.Lvo 13/04/2017, n. 66. D.I. n. 182 del 29 dicembre 2020. Adozione del modello nazionale di piano educativo individualizzato e delle correlate linee guida, nonché modalità di assegnazione delle misure di sostegno agli alunni con disabilità, ai sensi dell'articolo 7, comma 2-ter del decreto legislativo 13 aprile 2017, n. 66. Nota MI n. 40 del 13 gennaio 2021. Modalità per l'assegnazione delle misure di sostegno e nuovo modello di PEI ai sensi dell'Art. 7, comma 2-ter del decreto legislativo 66/2017. Decreto del Ministro dell'istruzione 29 dicembre 2020, n.182 D. n. 75 del 26 gennaio 2021. Misure di accompagnamento delle istituzioni scolastiche alle nuove modalità di inclusione previste dal decreto legislativo 13 maggio 2017, n. 66 e alle disposizioni contenute nel Decreto interministeriale 29 dicembre 2020, n 182. Nota MI n. 2044 del 17 settembre 2021. Sentenza n. 9795/2021 del 14/09/2021, TAR Lazio. Indicazioni operative per la redazione dei PEI per l'a.s.2021/2022. Nota 71 del 21 gennaio 2022 - Attività in presenza alunni con disabilità o con bisogni educativi speciali. Nota 22 mar 2022. Interventi a favore degli studenti con disabilità certificata. 29 luglio 2022. Ministro per le disabilità. Misure di sostegno per le persone con disabilità https://disabilita.governo.it/it/. Translation available in Supplementary material.

^4^D.Lvo n. 286 del 25 luglio 1998. Testo unico delle disposizioni concernenti la disciplina dell'immigrazione e norme sulla condizione dello straniero. DPR n. 394 del 31 agosto 1999. Regolamento recante norme di attuazione del TU delle disposizioni concernenti la disciplina dell'immigrazione e norme sulla condizione dello straniero, a norma dell'art. 1, c. 6, del D.Lvo 286/1998, (art. 45). Legge n. 189 del 30 luglio 2002. Modifica alla normativa in materia di immigrazione e di asilo (c.d. Legge Bossi-Fini). CM n. 24 del 1 marzo 2006. Trasmissione delle “Linee guida per l'accoglienza e l'integrazione degli alunni stranieri 2006”. Comunicato stampa MIUR 23 ottobre 2007. Presentazione del Documento “La via italiana per la scuola interculturale e l'integrazione degli alunni stranieri”, a cura dell'Osservatorio nazionale per l'integrazione degli alunni stranieri e per l'educazione interculturale. CM n. 2 dell'8 gennaio 2010. Indicazioni e raccomandazioni per l'integrazione di alunni con cittadinanza non italiana. CM n. 465 del 27 gennaio 2012. Studenti con cittadinanza non italiana iscritti a classi di istituti di istruzione secondaria di secondo grado. Esami di Stato. Nota MIUR n. 4233 del 19 febbraio 2014. Trasmissione delle “Linee guida per l'accoglienza e l'integrazione degli alunni stranieri 2014” (con errata corrige del 19/05/2014). Legge n. 107 del 13 luglio 2015. Riforma del sistema nazionale di istruzione e formazione e delega per il riordino delle disposizioni legislative vigenti (in part. art. 1 co. 7 lett. r). Nota MIUR n. 5535 del 9 settembre 2015. Trasmissione del documento “Diversi da chi? Raccomandazioni per l'integrazione degli alunni stranieri e per l'intercultura. Dipartimento per il sistema educativo di istruzione e formazione. 15 aprile 2022. Accoglienza scolastica per gli studenti ucraini. Indicazioni operative. Translation available in Supplementary material.

^5^D. Lgs. 66/2017 - Norme per la promozione dell'inclusione scolastica degli studenti con disabilità. Il Decreto legislativo 13 aprile 2017. Translation available in Supplementary material.

^6^Chieppa, M., and Migrazioni e disabilità, A. (2019). Un approccio intersezionale per una scuola plurale. *Educazione Interculturale. Teorie, ricerche, pratiche*. 17, 1. Available online at: https://rivistedigitali.erickson.it/educazione-interculturale/archivio/vol-17-n-1-2.
