# Supplementary material for: Disability and migrants: A double educational challenge for an inclusive and plural school
Source: Front Psychol. 2022 Nov 23;13:1017841. doi: 10.3389/fpsyg.2022.1017841 (PMC9728097; doi:10.3389/fpsyg.2022.1017841)
Supplement: Supplementary file 1 [file Presentation_1.pdf]

Note 3 Translation

Law n. 118 of 30 March 1971. Conversion into law of D.L. 30 January 1971, n. 5 and new norms in favor of the mutilated and disabled civilians. Act No. 517 of 4 August 1977. Rules on the assessment of pupils and the abolition of remedial examinations, as well as other rules for modifying the school system. Law No. 104 of 5 February 1992. Framework Law on Assistance, Social Integration and the Rights of Disabled Persons. DPR No. 24 February 1994. Act of guidance and coordination relating to the tasks of local health units in relation to handicapped pupils. DPR No. 323 of 23 July 1998. Regulation governing the final state examinations of upper secondary education courses pursuant to art. 1 of the law 10 December 1997 n. 425. MIUR note n. 4798 of 27 July 2005. Programming of the school integration of disabled pupils by the Educational Institutions - School year 2005-2006. OM No. 90 of 21 May 2001. Rules for the conduct of examinations and examinations in state and non-state primary, middle and upper secondary schools - School year 2000-2001, in part. Art. 15. C.M. No. 125 of 20 July 2001. Certificazione for students with disabilities. DPCM n. 185 of 23 February 2006. Regulation laying down detailed rules and criteria for the identification of the pupil as a person with a disability, pursuant to Article 35, paragraph 7, of Law No. 289 of 27 December 2002. MIUR Note No. 4274 of 4 August 2009. Transmission of the "Guidelines for school integration of pupils with disabilities". D.Lvo n. 66 of 13 April 2017. Rules for the promotion of school inclusion of students with disabilities, in accordance with Article 1, cc. 180 and 181, letter (c), of Law No. 107 of 13 July 2015. MIUR Note No. 1153 of 4 August 2017. Clarifications on the effective date of the terms of application of the indications of Legislative Decree No. 66/2017. MIUR Note No. 15578 of 8 August 2017. Clarifications on the starting date of the terms of application of the D.L. Decree n.66/2017. Clarification. D.Lvo n. 96 of 7 August 2019. Supplementary and corrective provisions to Legislative Decree 13/04/2017, n. 66. D.I. n. 182 of 29 December 2020. Adoption of the national model of individualized educational plan and related guidelines, as well as how to allocate support measures to pupils with disabilities, pursuant to Article 7, paragraph 2-ter of Legislative Decree no. 66 of 13 April 2017. Note No. 40 of 13 January 2021. Procedures for the allocation of support measures and new model of EIP pursuant to Art. 7, paragraph 2-ter of Legislative Decree 66/2017. Decree of the Minister of Education 29 December 2020, n.182 D. n. 75 of 26 January 2021. Measures to accompany educational institutions to the new methods of inclusion provided for by Legislative Decree No. 66 of 13 May 2017 and the provisions contained in the Ministerial Decree No 182 of 29 December 2020. Note MI No. 2044 of 17 September 2021. Judgment No. 9795/2021 of 14/09/2021, TAR Lazio. Operational guidelines for the drafting of the EIP for the year 2021/2022. Note 71 of 21 January 2022 - Activities with pupils with disabilities or special educational needs. Note 22 Mar 2022. Interventions for students with certain disabilities. 29 July 2022. Minister for Disability. Support measures for people with disabilities. https://disabilita.governo.it/it/.

Note 4 Translation

D.Lvo n. 286 of 25 July 1998. Consolidated text of the provisions concerning the regulation of immigration and rules on the status of foreigners. DPR No. 394 of 31 August 1999. Regulation laying down rules for the implementation of the TU of the provisions concerning the regulation of immigration and rules on the status of foreigners, pursuant to art. 1, c. 6, of D.Lvo 286/1998, (art. 45). Law no. 189 of 30 July 2002. Change to the legislation on immigration and asylum (c.d. Bossi-Fini Law). CM n. 24 of 1 March 2006. Transmission of the "Guidelines for the reception and integration of foreign pupils 2006". Press release MIUR 23 October 2007. Presentation of the Document "La via italiana per la scuola interculturale e l'integrazione degli alunni stranieri", by the National Observatory for the integration of foreign pupils and intercultural education. CM n. 2 of 8 January 2010. Indications and recommendations for the integration of students with non-Italian citizenship. CM n. 465 of 27 January 2012. Students with non-Italian citizenship enrolled in classes of secondary education institutions. State examinations. MIUR note no. 4233 of 19 February 2014. Transmission of the "Guidelines for the reception and integration of foreign students 2014" (with incorrect corrige of 19/05/2014). Law No. 107 of 13 July 2015. Reform of the national system of education and training and delegation for the reorganization of existing legislation (in part. art. 1 co. 7 lit. r). Note MIUR n. 5535 of 9 September 2015. Transmission of the document "Other than who? Recommendations for the integration of foreign pupils and for interculture. Department for education and training system. 15 April 2022. School reception for Ukrainian students. Operational directions.

Note 5 Translation

D. Lgs. 66/2017 - Norms for the promotion of school inclusion of students with disabilities. Legislative Decree 13 April 2017.
